# The Beneficial Effects of Combination Therapy With SGLT-2 Inhibitors and GLP-1 Receptor Agonists in Type 2 Diabetes Mellitus

**DOI:** 10.2174/0115733998419380251126073540

**Published:** 2026-01-13

**Authors:** Aimilianos Kalogeris, Ignatios Ikonomidis, Konstantinos G. Kyriakoulis, George Pavlidis, John Thymis, Konstantinos Katogiannis, Aikaterini Kountouri, Loukia Pliouta, Sotirios Pililis, Emmanouil Korakas, Vaia Lambadiari

**Affiliations:** 1 National and Kapodistrian University of Athens, School of Health Sciences, Department of Medicine, Athens, Greece

**Keywords:** Cardiovascular disease, combination therapy, GLP-1 receptor agonist, glycemic control, SGLT-2 inhibitors, type 2 diabetes mellitus

## Abstract

Type 2 diabetes mellitus is a chronic metabolic disorder associated with microvascular and macrovascular complications. Hyperglycemia and insulin resistance are core pathophysiological components of diabetes, linked to subclinical inflammation and persistent oxidative stress, which result in endothelial dysfunction and subsequent atherogenesis. Nowadays, several arrows in the diabetologist's quiver are available for the management of diabetes, providing flexibility and the ability to adopt a personalized approach tailored to each patient's needs. Two of the most commonly prescribed antidiabetic drugs are sodium-glucose cotransporter 2 inhibitors and glucagon-like peptide-1 receptor agonists. Both agents are associated with beneficial metabolic, cardio- and nephro-protective effects independent of their antidiabetic properties; thus, their indications have and are continuously expanding over and beyond the treatment of diabetes. Given that these two drug classes have different mechanisms of action, the use of their combination achieves a synergistic interaction and confers additional benefits. The aim of this review is to provide a summary of current knowledge regarding the use of these two drug classes, shed light on the available evidence on their combination, and discuss future perspectives on optimal therapeutic decisions in clinical practice. Sodium-glucose cotransporter 2 inhibitors and glucagon-like peptide-1 receptor agonists in combination therapy are a promising therapeutic duo and are expected to influence future guidelines and decision-making in everyday clinical practice.

## 1 INTRODUCTION AND BACKGROUND

Type 2 diabetes mellitus (T2DM) represents a complex and chronic metabolic disorder with multiple microvascular and macrovascular complications affecting almost all systems of the human body [1-4]. The underlying pathophysiological mechanisms are heterogeneous and include insulin resistance and hyperglycemia that are further associated with low-grade inflammation and chronic enhancement of oxidative stress, triggering endothelial dysfunction and promoting atherogenesis [1].

The treatment strategy for T2DM has impressively advanced from the initial guideline reports almost 20 years ago to the treatment algorithm implemented today [5, 6]. Back in 2006, the main therapeutic options included lifestyle interventions, metformin, sulfonylureas, glitazones, and insulin [5]; while novel drug classes for specific conditions (*e.g.*, high cardiovascular risk) are currently available [6]. Two of the most commonly prescribed antidiabetic drugs are sodium-glucose cotransporter 2 inhibitors (SGLT-2i) and glucagon-like peptide-1 (GLP-1) receptor agonists (GLP-1RA) [6]. SGLT-2i acts by blocking the reabsorption of glucose in the kidneys, resulting in increased glucose excretion in the urine; while GLP-1RA mimics the action of the natural GLP-1 hormone, which promotes insulin and reduces glucagon secretion [6]. Both drug classes have been linked to beneficial metabolic, cardio- and nephro-protective effects largely independent of their antidiabetic properties [6]. The combined use of these agents is expected to offer synergistic benefits across various cardiometabolic aspects, including glycemic control, weight loss, BP control, lipid profile regulation, cardiorenal outcomes, and other cardiovascular functional parameters (*e.g.*, central BP, echocardiographic indices, and others) [7-10].

The aim of this brief review is to provide an overview of current knowledge regarding the use of SGLT-2 inhibitors (SGLT-2i) and GLP-1 receptor agonists (GLP-1RA), highlight the available evidence on their combined use and the additional clinical benefits it may offer, and discuss future perspectives for optimizing therapeutic decisions in everyday clinical practice. To achieve this, a literature search was conducted in MEDLINE/PubMed using relevant keywords (*e.g.*, sodium-glucose cotransporter 2 inhibitors, SGLT-2i, glucagon-like peptide-1 receptor agonists, GLP-1RA, combination therapy) to identify eligible studies for inclusion and discussion.

## 2 REVIEW

### 2.1 Sodium-Glucose Cotransporter 2 Inhibitors

SGLT-2 inhibitors (SGLT-2i) belong to the family of gliflozins, originally developed as antidiabetic agents. In humans, the two most significant sodium-glucose cotransporters are SGLT-1 and SGLT-2. SGLT-1 is primarily expressed in the small intestine, heart, and skeletal muscles, whereas SGLT-2 is localized in renal tissue [11]. Approximately 90-97% of filtered glucose is reabsorbed *via* these cotransporters [12]. The antidiabetic effect of gliflozins is mediated through glycosuria resulting from the inhibition of renal glucose reabsorption by blocking SGLT-2 in the proximal tubules of the kidney, which also promotes sodium excretion [13]. SGLT-2i are considered moderate-intensity antidiabetic agents, as they reduce hemoglobin A1c by about 1%. Additionally, by inducing glycosuria-associated sodium excretion, they restore tubuloglomerular feedback, reduce intraglomerular pressure, and thereby exert nephroprotective effects [13].

Several preclinical animal studies have suggested that SGLT-2 inhibitors (SGLT-2i) may exert beneficial effects on endothelial function in both the microvascular and macrovascular systems. A recent study investigating the effects of dapagliflozin on endothelial dysfunction, assessed both *in vivo* (mice) and *in vitro* (human coronary artery endothelial cells), demonstrated that dapagliflozin effectively reduces cardiac microvascular damage and endothelial dysfunction during ischemia/reperfusion injury by inhibiting the XO-SERCA2-CaMKII-cofilin pathway [14]. Pretreatment with dapagliflozin attenuated luminal stenosis, endothelial swelling, and inflammation in the cardiac microvessels of mice [14]. Similarly, an experimental study with empagliflozin in mice suggested that it may mitigate cardiac microvascular ischemia/reperfusion injury *via* activation of the AMPKα1/ULK1/FUNDC1/mitophagy pathway [15]. Specifically, empagliflozin appeared to prevent reperfusion-induced microvascular damage through AMPKα1 activation and FUNDC1-dependent mitophagy, which normalized mitochondrial fusion, reduced endothelial oxidative stress, and ultimately improved microvascular structure and endothelial function [15].

Experimental studies in humans have demonstrated that SGLT-2 inhibitors (SGLT-2i) exert beneficial effects beyond their antidiabetic properties, including modulation of neurohormones such as the renin-angiotensin-aldosterone system, alterations in energy substrate utilization, and reductions in systemic inflammation and oxidative stress [16]. Accumulating evidence, primarily from cardiovascular safety trials, has not only confirmed the cardiovascular safety of SGLT-2i but also highlighted its significant pleiotropic effects. These findings paved the way for landmark trials that provided robust evidence supporting the pivotal role of SGLT-2i in the treatment of established cardiovascular disease, particularly heart failure, irrespective of diabetic status [17, 18].

The commonly prescribed SGLT-2 inhibitors (SGLT-2i) include dapagliflozin, empagliflozin, canagliflozin, and ertugliflozin. According to US Food and Drug Administration recommendations, four landmark clinical trials have investigated their cardiovascular effects: DECLARE-TIMI 58 for dapagliflozin [19], EMPA-REG OUTCOME for empagliflozin [20], CANVAS for canagliflozin [21], and VERTIS CV for ertugliflozin [22]. Sotagliflozin, a combined SGLT1/SGLT2 inhibitor, showed limited success in type 1 diabetes mellitus but demonstrated cardiovascular benefits similar to other SGLT-2i in individuals with T2DM [23, 24]. The initial results of these studies were highly promising, as all agents significantly reduced the risk of cardiovascular death and hospitalization for heart failure in diabetic patients [25, 26] (Fig. **1A**). Currently, empagliflozin and dapagliflozin have established roles in the treatment of heart failure, irrespective of T2DM status or ejection fraction category [27, 28].

Similarly, the initial cardiovascular safety trials suggested potential benefits of SGLT-2 inhibitors (SGLT-2i) on kidney outcomes in participants with chronic kidney disease (CKD) and albuminuria [25, 26]. Dedicated trials specifically designed to assess kidney outcomes in CKD patients, regardless of diabetes status, have also been conducted: DAPA-CKD for dapagliflozin [29], EMPA-KIDNEY for empagliflozin [30], and CREDENCE for canagliflozin [31]. As a result, after nearly two decades during which renin-angiotensin-aldosterone system blockers were the only treatment option for CKD, SGLT-2i have now been officially recognized as one of the key pillars in the management of CKD [32].

In addition to trials assessing the effects of SGLT-2 inhibitors (SGLT-2i) on major cardiovascular outcomes, several studies have evaluated their impact on surrogate markers. The EMPA-HEART CardioLink-6 randomized clinical trial demonstrated that empagliflozin reduced left ventricular mass, measured by magnetic resonance imaging, after six months in patients with T2DM, stable coronary artery disease, and preserved ejection fraction [33]. Beyond their antidiabetic effects, SGLT-2i have also shown beneficial metabolic effects. The EFFECT II trial investigated liver outcomes in 84 T2DM patients with non-alcoholic fatty liver disease (previously termed metabolic dysfunction-associated steatotic liver disease) [34]. It found that dapagliflozin, alone or combined with omega-3 carboxylic acids, significantly reduced liver fat content on magnetic resonance imaging, while dapagliflozin monotherapy decreased hepatocyte injury biomarkers and fibroblast growth factor over 12 weeks [34]. Another randomized trial in 56 T2DM patients reported that canagliflozin did not alter intrahepatic triglyceride content assessed by magnetic resonance spectroscopy but improved hepatic insulin sensitivity, insulin secretion, and clearance over 24 weeks [35].

### 2.2 Glucagon-Like Peptide-1 Receptor Agonists

GLP-1 is a peptide hormone secreted by intestinal epithelial L-cells, primarily in response to glucose and fat. Its main role is as an incretin, regulating postprandial glucose homeostasis by enhancing glucose-stimulated insulin secretion from pancreatic β-cells, inhibiting glucagon release, and slowing gastric emptying [36, 37]. Experimental studies also show that GLP-1 promotes β-cell neogenesis, inhibits apoptosis, and reduces glucagon secretion from α-cells [38]. Additionally, GLP-1 contributes to satiety in the hypothalamus, increases glucose uptake in skeletal muscles, and delays gastric emptying [26].

Several GLP-1 receptor agonists (GLP-1RA), including exenatide, liraglutide, lixisenatide, dulaglutide, and semaglutide, have established their role in T2DM management [39]. These drugs improve glycemic control while reducing body weight and systolic blood pressure [40-42]. Furthermore, liraglutide, dulaglutide, and semaglutide have received US Food and Drug Administration approval for reducing cardiovascular risk in patients with T2DM and established cardiovascular disease [43].

The cardiovascular benefits of GLP-1 receptor agonists (GLP-1RA) have been extensively studied in recent years (Fig. **1B**). In 2016, the ELIXA trial evaluated the short-acting agent lixisenatide in T2DM patients with a history of acute coronary syndrome, demonstrating cardiovascular safety but no additional cardiovascular benefit [44]. Similar neutral results were reported in the PIONEER 6 (oral semaglutide) [45] and EXSCEL (exenatide) trials [46]. Subsequently, results from larger trials have highlighted the potential of GLP-1RA to reduce cardiovascular risk in patients with T2DM. The LEADER trial showed that liraglutide reduced the risk of major adverse cardiovascular events-including nonfatal stroke, nonfatal myocardial infarction, and cardiovascular death-by 13%, with a 22% reduction in cardiovascular morbidity, a 15% reduction in all-cause morbidity, and a 22% reduction in diabetic kidney disease [47]. In the SUSTAIN 6 trial, 2,735 high cardiovascular risk T2DM patients treated with semaglutide for 104 weeks exhibited a lower risk of cardiovascular death, nonfatal myocardial infarction, or stroke compared to placebo, confirming the noninferiority of semaglutide [48]. The HARMONY trial demonstrated that albiglutide was superior to placebo in reducing major adverse cardiovascular events in patients with T2DM and cardiovascular disease [49]. Finally, in the REWIND trial, dulaglutide showed particularly promising results in middle-aged and older patients with T2DM, whether they had previous cardiovascular disease or cardiovascular risk factors [50].

A meta-analysis evaluating cardiovascular and kidney outcomes across seven GLP-1 receptor agonists (GLP-1RA), encompassing a total of 56,004 T2DM participants, found that GLP-1RA reduced major adverse cardiovascular events by 14%, all-cause mortality by 12%, heart failure hospitalizations by 11%, and the composite kidney outcome by 21%, without a significant increase in adverse events [51]. The kidney-protective effects of GLP-1RA have also been confirmed in recent dedicated studies, such as the FLOW trial [52, 53]. Consequently, GLP-1RAs have emerged as the fourth pillar in the management of diabetic chronic kidney disease (CKD) [54].

Beyond their metabolic and cardiovascular benefits, GLP-1 receptor agonists (GLP-1RA) have been investigated for potential neuroprotective effects, as suggested by reductions in stroke rates in primary prevention settings. A systematic review and meta-analysis of five multicenter, randomized, placebo-controlled trials (ELIXA, LEADER, SUSTAIN, EXSCEL, and HARMONY) reported a 13% reduction in stroke risk among participants receiving GLP-1RA; however, this protective effect was not observed when fatal stroke events were included [26, 55]. GLP-1RA has also shown benefits in endothelial function, which links microvascular and macrovascular complications of T2DM. Various studies have demonstrated vasodilatory effects, improved blood flow, and favorable cardiac outcomes related to coronary ischemia [56, 57]. It remains unclear whether these endothelial benefits are mediated directly *via* endothelial GLP-1 receptors, as many studies do not adjust for GLP-1RA-induced increases in insulin secretion or reductions in blood glucose. Therefore, the observed improvements in endothelial function may involve indirect mechanisms [57].

A new era has emerged with the introduction of tirzepatide, a novel dual glucose-dependent insulinotropic polypeptide (GIP)/GLP-1 receptor agonist (GLP-1RA). In the SURPASS-1 trial, tirzepatide demonstrated significant improvements in glycemic control and body weight in participants with T2DM, while maintaining a safety profile comparable to that of GLP-1RA [58]. Given its pronounced effects on body weight, the SURMOUNT-1 trial assessed tirzepatide in non-diabetic adults with obesity and reported substantial and sustained weight reduction [59]. Additional studies have highlighted the potential benefits of tirzepatide in various weight-related conditions, including sleep apnea and heart failure with preserved ejection fraction [60, 61]. Future research is expected to clarify the cardiometabolic and kidney effects of tirzepatide, guiding optimal therapeutic strategies [6].

### 2.3 Combination Therapy with GLP-1RA and SGLT-2i

Both SGLT-2 inhibitors (SGLT-2i) and GLP-1 receptor agonists (GLP-1RA) are drug classes that provide benefits beyond glycemic control in T2DM. Because their mechanisms of action differ, combining these two classes may produce synergistic effects and confer additional advantages [62]. The potential benefits of SGLT-2i and GLP-1RA combination therapy are summarized in Fig. (**2**), including improvements in glycemic control, body weight, blood pressure, lipid profile, renal outcomes, and other cardiovascular indices such as central blood pressure, pulse wave velocity (PWV), echocardiographic parameters, neurohumoral and mitochondrial function, and endothelial glycocalyx thickness. The concept of SGLT-2i/GLP-1RA combination therapy has recently attracted considerable interest, with several (mostly observational) studies comparing combination therapy to monotherapy (either SGLT-2i or GLP-1RA) reporting superior outcomes across various clinical settings [7-10].

Regarding glycemic control, a prospective, placebo-controlled study was conducted in 695 T2DM patients who were poorly controlled on maximum doses of metformin [63]. Participants received 28 weeks of treatment with either exenatide alone (n = 231), dapagliflozin alone (n = 233), or a combination of dapagliflozin and exenatide (n = 231) [63]. Starting from a baseline hemoglobin A1c of 9.3%, reductions of 1.6%, 1.4%, and 2.0% were observed in the exenatide, dapagliflozin, and combination groups, respectively. Notably, the magnitude of hemoglobin A1c reduction is proportional to baseline levels, with higher initial values resulting in greater decreases [64].

SGLT-2 inhibitors (SGLT-2i) promote weight loss by increasing urinary glucose excretion, which translates to a caloric loss of approximately 4 calories per gram of glucose. However, compensatory increases in food intake can reduce the net weight loss compared to the expected effect based on glucosuria alone [64]. GLP-1 receptor agonists (GLP-1RA), in contrast, reduce appetite, making SGLT-2i/GLP-1RA combination therapy particularly effective for weight reduction. In the CANVAS trial, adding canagliflozin to a GLP-1RA resulted in an average body weight reduction of 3.6 kg [65]. Similarly, in the DURATION-8 trial, weight loss with exenatide monotherapy was 1.54 kg and with dapagliflozin monotherapy 2.19 kg, both lower than the 3.41 kg reduction observed with the exenatide/dapagliflozin combination [63]. Two smaller retrospective studies reported weight reductions of 4.6 kg and 3.1 kg when canagliflozin [66] or dapagliflozin [67] were added to GLP-1RA therapy, respectively. Additionally, both drug classes have shown promising effects in reducing visceral fat [68, 69], while GLP-1RA have been particularly effective in decreasing hepatic fat in patients with T2DM and nonalcoholic fatty liver disease or nonalcoholic steatohepatitis [70].

The effects of SGLT-2 inhibitors (SGLT-2i) on arterial hypertension have been widely studied. By inducing natriuresis, these drugs reduce intravascular volume and alter intrarenal hemodynamics, leading to decreases in both systolic and diastolic blood pressure (BP) [71]. GLP-1 receptor agonists (GLP-1RA) similarly produce modest BP reductions, likely through mild natriuresis and vasodilation [72]. Because these drugs lower BP *via* different mechanisms, their combination is expected to have additive effects. In the DURATION-8 trial, systolic BP decreased by 4.1 mmHg with exenatide/dapagliflozin combination therapy, compared to 1.3 mmHg and 1.8 mmHg with exenatide and dapagliflozin monotherapy, respectively [63]. Both SGLT-2i and GLP-1RA enhance renal sodium excretion. SGLT-2 facilitates 1:1 glucose and sodium reabsorption in the proximal tubule [73], so SGLT-2i promotes increased urinary excretion of both sodium and glucose, reducing intravascular volume and lowering BP [63]. The mechanisms underlying GLP-1RA-induced sodium excretion are less clear but may involve vasodilation or direct tubular effects.

GLP-1 receptor agonists (GLP-1RA) influence lipid metabolism by reducing intestinal chylomicron production and secretion, as well as suppressing postprandial triglycerides and apolipoprotein B48 levels [74]. In contrast, SGLT-2 inhibitors (SGLT-2i) have minimal effects on plasma lipid profiles, causing only slight increases in low-density lipoprotein cholesterol (~3.5%) and modest decreases in triglycerides (~5-10%) [75]. Consistently, the DURATION-8 trial reported no significant differences in plasma lipid levels among dapagliflozin monotherapy, exenatide monotherapy, and the dapagliflozin/exenatide combination therapy groups [63]. Additionally, a meta-analysis of the LEAD trials found that liraglutide reduced low-density lipoprotein cholesterol by 7.73 mg/dL, total cholesterol by 5.03 mg/dL, and triglycerides compared with standard treatment [76].

A 12-month randomized study in 160 patients examined the effects of combining SGLT-2 inhibitors (SGLT-2i) and GLP-1 receptor agonists (GLP-1RA) on vascular and cardiac function, focusing on endothelial glycocalyx thickness, pulse wave velocity (PWV), central systolic blood pressure, global left ventricular longitudinal, circumferential, and radial strain, and myocardial work using speckle-tracking imaging [77]. Both drug classes showed beneficial effects individually, but the combination therapy produced even greater improvements. The combination led to larger reductions in glycocalyx thickness, PWV, and central systolic BP compared with insulin, while hemoglobin A1c reductions were similar across groups (*p* < 0.05). GLP-1RA alone increased global work index by 12.7%, whereas the combination with SGLT-2i increased it by 17.4%, compared to 3.1% with insulin and 2% with SGLT-2i monotherapy. Additionally, SGLT-2i or GLP-1RA combined with SGLT-2i resulted in greater reductions in PWV and central/brachial systolic BP than insulin or GLP-1RA alone (Fig. **3**). The most pronounced benefits of combination therapy were observed in patients with left ventricular ejection fraction below 55%. A secondary analysis of this study demonstrated that the combination also improved neurohumoral markers (N-terminal pro-brain natriuretic peptide, growth differentiation factor 15) and mitochondrial activation factors (mitochondrial-derived peptide-c, 2,2′-azino-bis(3-ethylbenzothiazoline-6-sulfonic acid)) more effectively than either treatment alone or insulin [78]. These findings support the beneficial role of SGLT-2i/GLP-1RA combination therapy in early cardiovascular disease, prior to the onset of overt heart failure or ischemia. Given that both SGLT-2 inhibitors (SGLT-2i) and GLP-1 receptor agonists (GLP-1RA) are now established pillars in the management of diabetic chronic kidney disease (CKD) [54], their combination is expected to provide additional benefits for renal outcomes. Several post-hoc analyses and observational studies support this hypothesis [79, 80]; however, further well-designed trials are required to confirm these effects.

## 3 FUTURE PERSPECTIVES

SGLT-2 inhibitor (SGLT-2i) and GLP-1 receptor agonist (GLP-1RA) combination therapy clearly provides additive benefits compared to either drug alone. Importantly, these effects occur without increasing the risk of hypoglycemia or other adverse events, largely independent of their antidiabetic properties, and complement existing treatments such as renin-angiotensin-aldosterone system blockers [6].

Looking ahead, the future of these therapies is promising. First, the initial signals of benefit need confirmation through large, well-designed randomized controlled trials, ideally with multiple treatment arms and diverse populations beyond patients with diabetes. Although challenging, such trials would provide the highest-quality evidence for the effects of SGLT-2i/GLP-1RA combination therapy on hard clinical endpoints. Second, single-pill combinations of these agents are expected to improve adherence and overall outcomes. This is particularly relevant because most GLP-1RA regimens are currently subcutaneous; the future availability of oral GLP-1RA in combination with SGLT-2i could facilitate wider and more convenient use. Finally, preclinical data suggest potential benefits of combining SGLT-2i with newer, more potent agents such as tirzepatide, a dual GIP/GLP-1RA [81], and further studies in humans are eagerly awaited.

## CONCLUSION

The combination of SGLT-2 inhibitors (SGLT-2i) and GLP-1 receptor agonists (GLP-1RA) has been shown to improve glycemic control and reduce cardiovascular risk in patients with T2DM. This dual therapy can provide greater benefits than either drug alone, without additional safety concerns. Prescription of combination therapy should be individualized, taking into account factors such as age, comorbidities, and medication tolerance, and its use is expected to increase over time. Patients with T2DM should be closely monitored by healthcare providers who are well-informed about contemporary therapeutic options to ensure the most appropriate treatment plan. Future research is warranted to fully explore the therapeutic potential of SGLT-2i/GLP-1RA combination therapy, both in T2DM and potentially in other populations.

## Figures and Tables

**Fig. (1):**
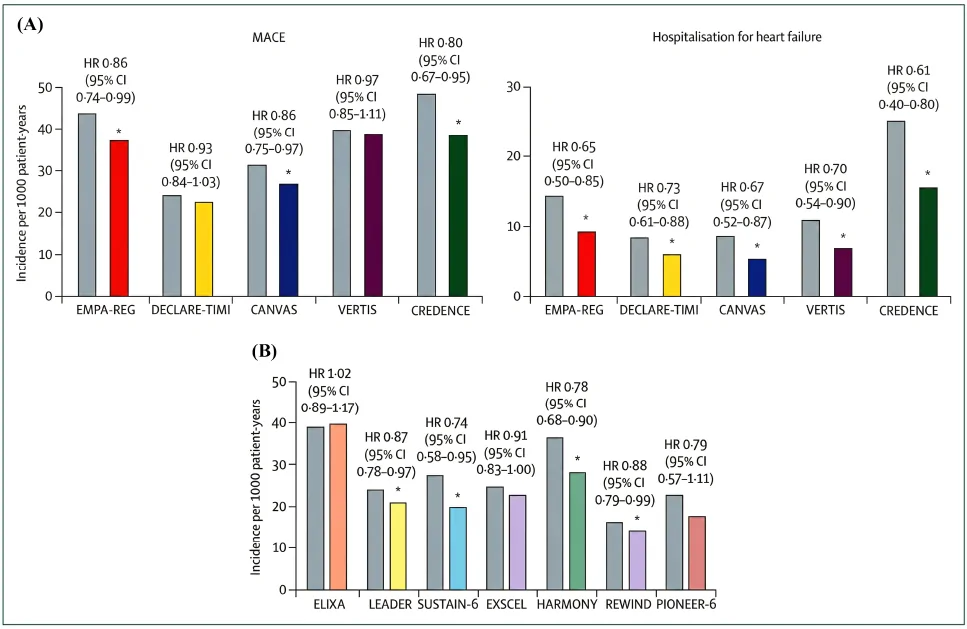
Cardiovascular outcomes from the key cardiovascular outcome trials. Cardiovascular outcomes from the key cardiovascular outcome trials with **(A)** SGLT-2i *versus* placebo (grey bars) and **(B)** GLP-1RA *versus* placebo (grey bars). Data are shown as incidence per 1000 patient years. HR=hazard ratio. MACE=major adverse cardiovascular events. *<0·05 *versus* placebo. Caution should be used when comparisons are made between individual trials due to differences in trial design and baseline characteristics. Reprinted from The Lancet, Volume 398, Emily Brown, Hiddo J. L. Heerspink, Daniel J. Cuthbertson, John P. H. Wilding. SGLT2 inhibitors and GLP-1 receptor agonists: established and emerging indications, Pages 262-276, Copyright 2021, with permission from Elsevier [26].

**Fig. (2):**
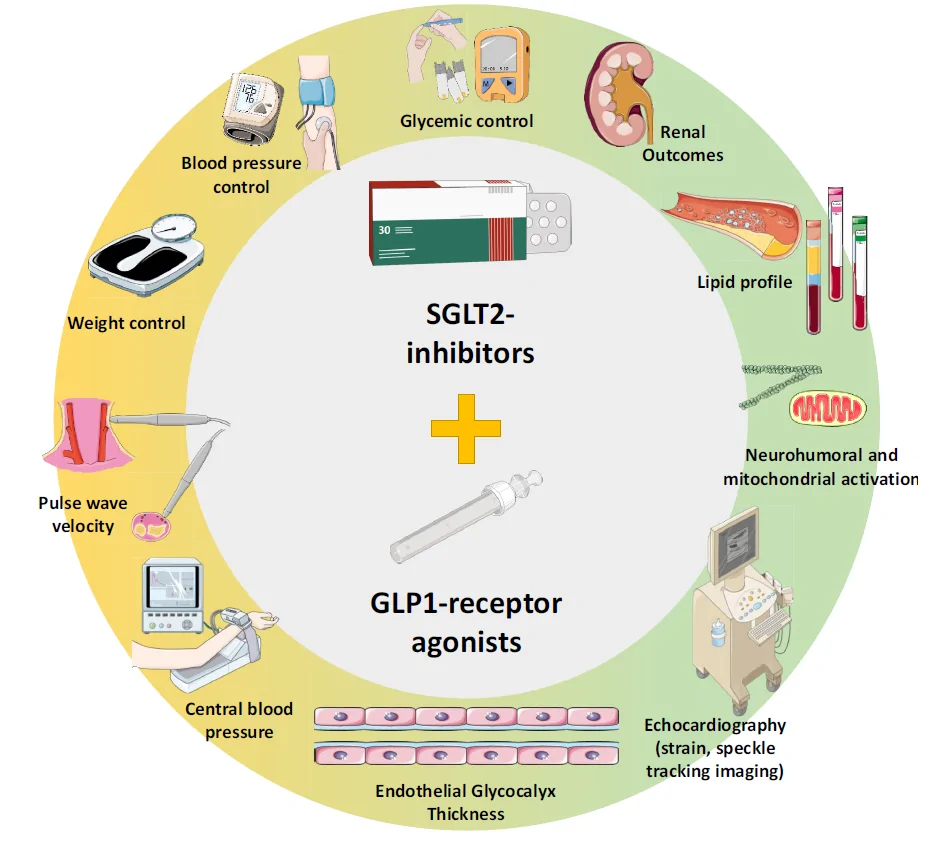
Graphical summary of the potential beneficial effects of SGLT-2i and GLP1-RA combination therapy.

**Fig. (3):**
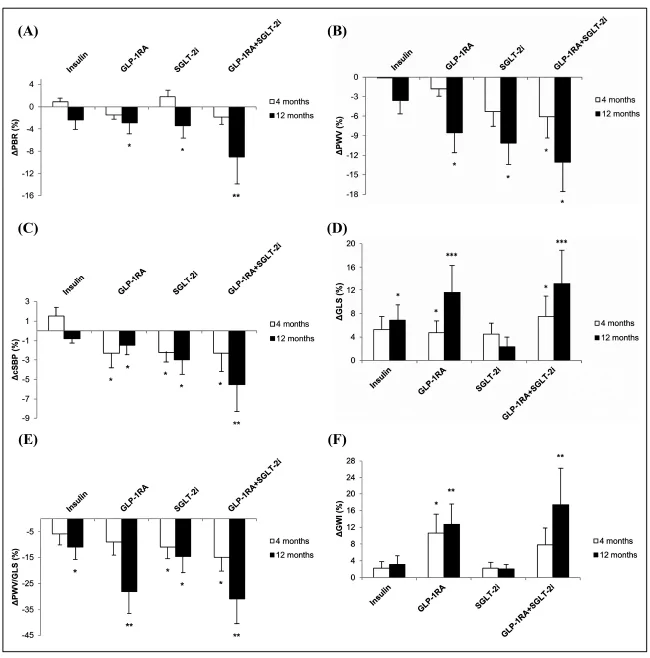
Effects of SGLT-2i and GLP1-RA combination therapy on indices of vascular and cardiac function. Percentage changes (Δs) from baseline in perfused boundary region (PBR) (**A**), pulse wave velocity (PWV) (**B**), central systolic blood pressure (cSBP) (**C**), left ventricular global longitudinal strain (GLS) (**D**), PWV/GLS ratio (**E**), and myocardial global work index (GWI) (**F**) at 4 and 12 months in the 4 study groups. Data shown are means ± SD. *p<0.05, **p<0.01, ***p<0.001 *vs.* baseline. Reproduced from Ikonomidis *et al.*, Journal of the American Heart Association; published by AHA/ASA Journals, 2020 under the Creative Commons CC BY-NC-ND 4.0 license [77].

## LIST OF ABBREVIATIONS

BP: Blood Pressure
CKD: Chronic Kidney Disease
GIP: Glucose-dependent Insulinotropic Polypeptide
GLP-1: Glucagon-Like Peptide-1
GLP-1RA: Glucagon-Like Peptide-1 Receptor Agonists
PWV: Pulse Wave Velocity
SGLT-2i: Sodium-Glucose Cotransporter-2 inhibitors
T2DM: Type 2 Diabetes Mellitus
